# Effects of Resveratrol on Redox Status, Jejunal Injury, and Mitochondrial Function in Intrauterine Growth-Retarded Weaned Piglets

**DOI:** 10.3390/ani15030290

**Published:** 2025-01-21

**Authors:** Kang Cheng, Jinxiu Yao, Zhihua Song, Jin Huang, Hongyue Zhao, Ranya Yang, Yao Meng, Jinrong Wang, Yong Zhang

**Affiliations:** 1 Guangzhou Tanke Bio-Tech Co., Ltd., Guangzhou 510896, China; 2 School of Biological Engineering, Henan University of Technology, Zhengzhou 450001, China; yao19838648990@163.com (J.Y.); huangjin@haut.edu.cn (J.H.); 15938708890@163.com (R.Y.); wangjr@haut.edu.cn (J.W.); 3 School of International Education, Henan University of Technology, Zhengzhou 450001, China; zhihuasong@126.com (Z.S.); zhaohongyue1005@163.com (H.Z.); 231170400521@stu.haut.edu.cn (Y.M.)

**Keywords:** intrauterine growth retardation, jejunal injury, resveratrol, mitochondrial function

## Abstract

This study investigated the effects of resveratrol (RSV) on redox status, jejunal injury, and mitochondrial function in intrauterine growth-retarded (IUGR) weaned piglets. In total, 12 male normal birth weight (NBW) newborn piglets and 24 male IUGR newborn piglets were selected. They were weaned at 26 days of age and then divided into three treatments in a three-week trial: (1) NBW piglets fed a basal diet; (2) IUGR piglets fed a basal diet; (3) IUGR piglets fed a basal diet supplemented with 300 mg/kg RSV. Compared with NBW piglets, IUGR piglets showed decreased growth performance, altered redox status, impaired jejunal morphology, tight junction protein expression, energy production, and mitochondrial oxidative phosphorylation. RSV enhanced antioxidant defense capacity and improved jejunal morphology, ATP generation, and mitochondrial complex I content in IUGR weaned piglets. Dietary RSV supplementation alleviated the IUGR-induced jejunal injury in weaned piglets probably by improving redox status and mitochondrial function, suggesting that RSV has the potential to be a dietary intervention in the regulation of intestinal injury in IUGR piglets.

## Simple Summary

Intestinal injury and dysfunction induced by intrauterine growth retardation (IUGR) may contribute to poor growth rates and increased mortality and morbidity in the postnatal life. Resveratrol (RSV), a natural polyphenol, is a promising therapeutic and/or preventive treatment for intestinal disorders. Our study showed that RSV alleviated the IUGR-induced jejunal injury in weaned piglets, probably by improving redox status and mitochondrial function.

## 1. Introduction

Intrauterine growth retardation (IUGR), a common complication of pregnancy, refers to the restricted growth and development of the mammalian embryo/fetus or its organs during gestation, threatening animal production and human health [1]. The pig is a species of mammalian animal with multiple pregnancies and has a 15% to 20% incidence of IUGR as a result of various perinatal insults [2]. The IUGR not only reduces the perinatal survival rate of the newborn piglets but also exerts a permanent negative impact on the health status, growth, and development of pigs after birth [3,4]. As the small intestine is an important organ for digestion, absorption, and immunity, intestinal injury and dysfunction by IUGR may contribute to poor growth rates and increased mortality and morbidity in the postnatal life. Previous studies have shown that the impaired intestinal morphology, suppressed digestive enzyme activities, increased apoptosis of enterocytes, decreased expression of tight junction proteins, and altered redox and immune status were found in IUGR individuals in comparison with healthy ones [5,6,7]. Mitochondrial dysfunction may be a potential mechanism of intestinal disorder in IUGR [4]. Mitochondrial function is particularly important in the constantly energy-demanding enterocytes, where mitochondria produce over 90% of cellular ATP. Mitochondria also emerge as critical integrators of other essential cellular processes, such as cell death, involved in health and disease [8]. Therefore, it is of great significance that nutritional strategies to improve intestinal health and mitochondrial function in IUGR piglets are developed.

Resveratrol (RSV), a natural polyphenol, exists widely in grapes, polygonum cuspidatum, and peanuts [9,10]. It has many pharmacological effects, including antioxidation, anti-inflammation, anticancer effects, and lipid lowering [3,11]. Emerging evidence now suggests that RSV is a promising therapeutic and/or preventive treatment for intestinal-related diseases, such as inflammatory bowel disease [12], colorectal cancer [13], irritable bowel syndrome [14], and intestinal infectious diseases [15]. As for domestic animals, available findings have shown that dietary supplementation with RSV could improve intestinal damage, antioxidant status, and immunity in pigs challenged with different stressors such as deoxynivalenol [16,17] and ablactation [18]. In broilers exposed to diverse stress settings such as heat stress [19,20] and lipopolysaccharide [21], dietary RSV supplementation alleviated intestinal barrier dysfunction, oxidative stress, and immune imbalance. In suckling [4] and finishing [3] pigs suffering from IUGR, RSV could attenuate hepatic damage and mitochondrial dysfunction in part through improving mitochondrial biogenesis and redox status. However, the protective effects of RSV on the intestinal injury and mitochondrial function in IUGR weaned piglets are still unclear. Therefore, the present study was conducted to investigate the effects of RSV on redox status, intestinal injury, and mitochondrial function in IUGR weaned piglets, providing scientific basis for the future rational application of RSV in the feed of pigs with IUGR. In addition, this study may help to develop an effective method for treating intestinal diseases in humans with IUGR due to the high similarities between pigs and humans in anatomy, physiology, and nutrient metabolism.

## 2. Materials and Methods

### 2.1. Animals and Experimental Design

All animal procedures were approved by the Institutional Animal Care and Use Committee of Henan University of Technology (Zhengzhou, China) (Ethic Approval Code: HAUT20230301). Piglets with a birth weight (BW) near the mean value of the herd (within 0.5 standard deviation) were identified as the normal BW (NBW) piglets, and the IUGR piglets were defined as having a 2 standard deviation lower than the mean value of the herd [22]. At farrowing, 12 male NBW (1.59 ± 0.11 kg, Duroc × [Landrace × Yorkshire]) and 24 male IUGR (0.94 ± 0.06 kg) newborn piglets were obtained from 12 sows according to the selection criteria [3]. The piglets were weaned at 26 days of age, and then allocated to three groups with six replicates of two piglets per pen. Among them, the NBW piglets and half of the IUGR piglets were assigned to the NC and IC groups, respectively, and fed a basal diet. The remaining IUGR piglets were assigned to the IR group and fed with a basal diet supplemented with 300 mg/kg RSV (98% of purity, Shanghai Yuanye Bio-Technology Co., Ltd., Shanghai, China). The composition and nutrient amounts of the basal diet are given in Table S1. Piglets had free access to feed and water during the whole experimental period of 21 d. The initial body weight (IBW), final body weight (FBW), and feed intake (FI) of piglets during the experimental period were recorded, and then their body weight gain (BWG) and feed efficiency (FE) were calculated.

### 2.2. Sample Collection

At 47 days of age, six piglets per group were selected for blood sample collection after being fasted overnight. Blood was collected from the anterior vena cava of piglets and centrifuged at 2000× *g*, 4 °C for 20 min to obtain serum. The serum samples were stored at −80 °C until analysis. Subsequently, the selected piglets were anesthetized and sacrificed, and their abdominal cavities were immediately opened to harvest jejunum samples. A fraction of jejunum samples (approximately 1 cm) was fixed in 4% paraformaldehyde, while mucosal samples from the additional parts (approximately 20 cm) were snap frozen in liquid nitrogen and stored at −80 °C for subsequent analysis.

### 2.3. Jejunal Morphology Analysis

After fixation, jejunum samples were dehydrated, embedded, sectioned, deparaffinised, rehydrated, and stained with haematoxylin and eosin for morphological evaluation. Images were captured by a light microscope (RVL-100-G, ECHO Laboratories, San Diego, CA, USA). Villus height (VH), crypt depth (CD), and villus width (VW) were measured using the Image-Pro Plus 6.0 software (Media Cybernetics, San Diego, CA, USA). The VH:CD ratio and villus surface area (VSA) were calculated using the method as reported previously [7].

### 2.4. Evaluation of Redox Status in Jejunum and Serum

Reduced glutathione (GSH) and malondialdehyde (MDA) contents, as well as glutathione peroxidase (GPX) and total superoxide dismutase (T-SOD) activities in the jejunal mucosa, were measured using colorimetric kits by a spectrophotometer according to the instructions of the kits of the Nanjing Jiancheng Institute of Bioengineering (Nanjing, China). A hydroxylamine method was employed to analyze SOD activity [23]. The assay of GPX activity and GSH level was performed using 5, 5′-dithiobis (2-nitrobenzoic acid) as described previously [24]. The thiobarbituric acid method was performed for the determination of MDA content [25]. Protein content in the jejunal mucosa was determined with a bicinchoninic acid (BCA) protein assay kit purchased from the Nanjing Jiancheng Institute of Bioengineering (Nanjing, China).

### 2.5. Jejunal ATP and Mitochondrial Electron Transport Chain Complexes Contents Analysis

The ATP, complex I, complex III, and complex V contents in the jejunum were analyzed by commercial enzyme-linked immunosorbent assay kits following the manufacturer’s protocol (Jiangsu Meimian Industrial Co., Ltd., Yancheng, China).

### 2.6. Jejunal Gene Expression Analysis

Jejunal mucosal total RNA was extracted using the Total RNA Isolation Reagent (Vazyme, Nanjing, China). The RNA concentration, reverse transcription, and real-time PCR reactions of each sample were conducted based on the method described by Cheng et al. [26]. Briefly, total RNA concentration was measured by the NanoDrop 2000C spectrophotometer (Thermo Fisher Scientific, Waltham, MA, USA). Total RNA was reversed by the HiScript II Q RT Select SuperMix for qPCR(+gDNA wiper) (Vazyme, Nanjing, China). The quantitative real-time PCR reaction was performed using the ChamQ SYBR qPCR Master Mix kit (Vazyme, Nanjing, China). The expression of gene was normalized to glyceraldehyde-3-phosphate dehydrogenase by the 2^−ΔΔ^*^Ct^* method [27]. The primers used in this study are listed in Table 1.

### 2.7. Statistical Analysis

Results were analyzed using the SPSS statistical software (ver.27.0 for Windows, SPSS Inc., Chicago, IL, USA). The Shapiro–Wilk test was used to analyze the normality of data, while the homogeneity of variances was verified by the Levene test. Statistical differences between different treatments were determined via one-way analysis of variance and Tukey’s post hoc test for pairwise comparisons. Otherwise, significance was assessed using the non-parametric Kruskal–Wallis test and pairwise differences in rank sums. Differences were considered significant when *p* < 0.05. Results are expressed as means with standard errors.

## 3. Results

### 3.1. Growth Performance

Compared with the NBW piglets (Table 2), IUGR decreased (*p* < 0.05) IBW and FBW in piglets. The IUGR piglets showed a tendency to decrease BWG compared with the NBW piglets (*p* = 0.063). However, FI and FE in piglets among the three groups were not altered (*p* > 0.05).

### 3.2. Jejunal Morphology

**Figure 1:**
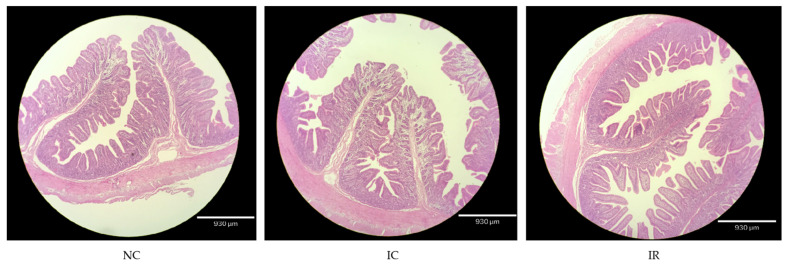
Representative images of jejunum sections stained by hematoxylin and eosin; scale bar = 930 μm. NC, piglets with normal birth weight fed a basal diet; IC, piglets with intrauterine growth retardation fed a basal diet; IR, piglets with intrauterine growth retardation fed a basal diet plus 300 mg/kg resveratrol.

In Table 3, VH, VH:CD ratio, and VSA were lower (*p* < 0.05) and CD was higher (*p* < 0.05) in the jejunum of the IC group than that of the NC group. Jejunal CD and VH:CD ratio in the IR group were improved compared with the IC group (*p* < 0.05). However, VW in the jejunum was not affected among the three groups (*p* > 0.05). The above results were validated from the histological appearance observation (Figure 1).

### 3.3. Jejunal Tight Junction Proteins mRNA Expression

Compared with the NC group (Table 4), the IC group showed a decrease in jejunal occludin (*OCLN*) mRNA expression (*p* < 0.05). No differences were found in the jejunal mRNA expression of zonula occludens 1, claudin 1, claudin 2, and claudin 3 in piglets among the three groups (*p* > 0.05).

### 3.4. Redox Status in Serum and Jejunum

Compared with the NC group (Table 5), IUGR increased MDA content in the serum and decreased the activities of T-SOD and GPX in the serum and T-SOD in the jejunum (*p* < 0.05). Conversely, RSV supplementation decreased MDA level in the serum and increased both T-SOD activity in the serum and T-SOD activity and GSH level in the jejunum in the IR group when compared with the IC group (*p* < 0.05). Serum GSH level and jejunal MDA content and GPX activity were not changed among the three groups (*p* > 0.05).

### 3.5. Jejunal ATP and Mitochondrial Electron Transport Chain Complexes Contents

Table 6 shows that jejunal ATP and complex I contents were lower in the IC group compared with the NC group (*p* < 0.05). The administration of RSV elevated jejunal ATP production and complex I content in the IR group when compared with the IC group (*p* < 0.05). The concentrations of complex III and V did not differ between the three groups (*p* > 0.05).

## 4. Discussion

Previous studies have reported that IUGR piglets’ growth slows down in their early growth stages [5,6], and their findings confirm our results, which exhibited that IUGR weaned piglets had lower IBW, FBW, and BWG. In this study, the impairment of growth performance in IUGR piglets could be attributed to intestinal injury and malfunction, which restricts nutrient utilization. However, in the present study, RSV administration did not alter growth performance in IUGR piglets. Similarly, in weaned [28] and finishing [29] pigs, RSV supplementation had no effect on growth performance. On the contrary, in broilers, the addition of RSV to the diet inhibited the lipopolysaccharide [21] and heat stress [19] induced decreased average daily gain. The effects of RSV on the growth performance may be related to the discrepancies in physiological conditions and animal species, and it needs more investigation.

Intestinal morphology is reflected by the VH, CD, VH:CD ratio, and VSA, which are considered to be key indicators of intestinal development and function, such as intestinal integrity and absorptive capability [30,31]. Similar to previous studies [5,6], IUGR significantly reduced the VH, VH:CD ratio, and VSA, and increased CD in the jejunum, which indicated that IUGR induced jejunal injury in weaned piglets. The administration of RSV mitigated IUGR-induced jejunal morphological damage indicated by the reduced CD and increased VH:CD ratio. Consistent with our results, previous studies have shown that dietary RSV ameliorated jejunal morphology impairment in pigs exposed to different stressors such as deoxynivalenol [17] and ablactation [32] evidenced by the increased VH and VH:CD ratio. Similar results were observed in broilers under heat stress [20]. These results suggest that RSV protects against intestinal injury in animals including IUGR piglets. In addition, the intestinal barrier determines intestinal homeostasis, which has been recognized as relevant to health and disease. Tight junction proteins between adjacent epithelial cells constitute the intestinal physical barrier, which can prevent the paracellular flux of luminal substances such as macromolecules and bacteria [33,34]. Occludin, an important extracellular component of the tight junction, serves to restrict epithelial permeability to low molecular mass molecules, and plays a critical role in maintaining intestinal barrier function [35]. In the present study, the down-regulated *OCLN* mRNA expression was found in the jejunum of IUGR piglets, which suggested that IUGR may disrupt the intestinal barrier function in weaned piglets and confirmed the results in the previous study [5]. Unfortunately, the jejunal tight junction proteins’ mRNA expression in IUGR weaned piglets was not altered by RSV administration. Similarly, Zhang et al. found that 300 mg/kg RSV supplementation had no effect on jejunal OCLN and ZO1 protein expression in the jejunum of weanling piglets from 21 to 28 days of age [18]. Chen et al. reported that 28-day-old weanling piglets supplemented with 150 and 300 mg/kg RSV for 42 days showed the increased jejunal *ZO1* mRNA expression and unchanged *OCLN* and *CLDN1* mRNA levels [32]. These results indicate that the effects of RSV on jejunal tight junction proteins may be associated with the dosage and duration of RSV, as well as the growth stage and physiological conditions of animal, which still requires experimental confirmation.

Redox status is a critical determinant of intestinal health and mitochondrial function [36]. Once disrupted, excessive free radical production or an impaired antioxidant defense system induced the high risk of intestinal diseases and mitochondrial dysfunction through oxidative damage to cellular biomolecules [5]. The MDA, a lipid peroxidation end product, is recognized as a reliable indicator of oxidative stress/redox imbalance in the organism [26]. The SOD and GPX can synergistically convert superoxide and hydrogen peroxide to carbon dioxide and water to protect the intestine and its mitochondria from oxidative damage [33]. The GSH, a non-enzymatic antioxidant, can directly quench reactive oxygen species and xenobiotics or indirectly participate in enzymatic antioxidant catalysis as the co-substrate of GPX [37]. Consistent with previous studies [5,6], our results showed that IUGR increased serum MDA level, and reduced serum T-SOD and GPX activities and jejunal T-SOD activity, suggesting that oxidative stress occurred in IUGR piglets. As expected, RSV improved IUGR-induced oxidative damage through enhancing serum T-SOD activity, jejunal T-SOD activity, and GSH level. Similarly, in IUGR suckling [4] and finishing [3] pigs, RSV alleviated the increased MDA content in the liver in part through enhancing Mn-SOD or T-SOD activities. In this study, in addition to its ability to enhance antioxidant defenses, the antioxidant property of RSV could be attributed to its hydroxyl group at 4′ and 5 position [38], reduced mitochondrial reactive oxygen species generation [39] and transcriptional or translational activation of antioxidant proteins and phase II detoxifying enzymes through different signaling pathways such as nuclear factor erythroid 2-related factor 2 protein [19] and sirtuin 1 (SIRT1) [40].

Enterocytes in the intestine have a high energy requirement as their roles involve rapid renewal of epithelium, defensive barriers, and the digestion and absorption of nutrients [5]. Mitochondria in enterocytes are the major sites of ATP production through oxidative phosphorylation mediated by the five complexes of the electron transport chain. Various endogenous and environmental stresses disrupt mitochondrial function (i.e., energy generation) by affecting critical processes in mitochondrial homeostasis like oxidative phosphorylation [41], including IUGR [4,5]. In the present study, we also found that IUGR induced the jejunal reduced ATP production and inhibited complex I content, indicating that the impaired energy generation and oxidative phosphorylation occurred and further contributed to intestinal damage. As expected, RSV improved jejunal ATP generation and complex I content in IUGR weaned piglets. Zhang et al. reported that RSV is effective in improving ATP depletion in the liver of IUGR suckling piglets through increasing complex I and complex V activities and preserving mitochondrial membrane potential, which is a key step in ATP synthesis [4]. In addition, the mechanism by which RSV mitigates the IUGR-induced mitochondrial oxidative phosphorylation inefficiency may be associated with its activation of SIRT1. Accumulating evidence indicates that SIRT1 can accelerate mitochondrial biogenesis by increasing the expression and deacetylation of peroxisome proliferation-activated receptor gamma coactivator-1 alpha (PGC1α) [42]. Mitochondrial biogenesis regulated by PGC1α is a process by which new mitochondria are produced from existing mitochondria, achieving ATP production. Chen et al. demonstrated that RSV improved diquat-induced intestinal injury in piglets by enhancing mitochondrial function via SIRT1 signaling [43]. In our study, RSV may also alleviate the IUGR-induced jejunal injury by affecting mitochondrial function, and the specific molecular mechanism needs to be further studied.

## 5. Conclusions

In conclusion, the results from the present study indicated that RSV alleviated the IUGR-induced jejunal injury in weaned piglets probably by improving redox status and mitochondrial function. Our data suggest that RSV has the potential to be a dietary intervention in the regulation of intestinal injury in IUGR weaned piglets. Although the price of RSV is currently high, it will eventually become an affordable and reasonable additive for livestock producers with the advancement of extraction processes, chemical synthesis, and biosynthesis technologies.

## Figures and Tables

**Table 1:** List of primers used in RT-qPCR.

Genes	Forward Primer Sequence (5′ → 3′)	Reverse Primer Sequence (5′ → 3′)
*ZO1*	AGAGGAAGCTGTGGGTAACG	TCACCGTGTGTTGTTCCCAT
*CLDN1*	ACAGGAGGGAAGCCATTTTCA	TTTAAGGACCGCCCTCTCCC
*CLDN2*	GGATCCTGCGGGACTTCTAC	TGGAGCGATTTCCTTGCAGT
*CLDN3*	GAGACCAGTCCACCCAGATG	AGGTTTCATGGTCCGTGCTG
*OCLN*	CAGGTGCACCCTCCAGATTG	ATGTCGTTGCTGGGTGCATA
*GADPH*	CCAAGGAGTAAGAGCCCCTG	AAGTCAGGAGATGCTCGGTG

*CLDN1*, claudin 1; *CLDN2*, claudin 2; *CLDN3*, claudin 3; *OCLN*, occludin; *GAPDH*, glyceraldehyde-3-phosphate dehydrogenase; *ZO1*, zonula occludens 1.

**Table 2:** Effects of resveratrol on growth performance in weaned piglets with intrauterine growth retardation.

Items	NC	IC	IR
Initial body weight, kg	7.72 ± 0.15 ^a^	5.93 ± 0.13 ^b^	5.79 ± 0.15 ^b^
Final body weight, kg	11.88 ± 0.07 ^a^	9.41 ± 0.22 ^b^	9.27 ± 0.30 ^b^
Body weight gain, kg	4.16 ± 0.09	3.48 ± 0.26	3.48 ± 0.18
Feed intake, kg	7.48 ± 0.15	6.61 ± 0.40	6.59 ± 0.22
Feed efficiency, kg/kg	0.56 ± 0.01	0.53 ± 0.02	0.53 ± 0.02

Results expressed as means and standard errors, *n* = 6. NC, piglets with normal birth weight fed a basal diet; IC, piglets with intrauterine growth retardation fed a basal diet; IR, piglets with intrauterine growth retardation fed a basal diet plus 300 mg/kg resveratrol. Significant differences between treatments are described by different letters in the form of superscripts, significant at *p* < 0.05.

**Table 3:** Effects of resveratrol on jejunal morphology in weaned piglets with intrauterine growth retardation.

Items	NC	IC	IR
Villus height, μm	331.36 ± 17.55 ^a^	263.10 ± 3.40 ^b^	305.23 ± 26.14 ^ab^
Crypt depth, μm	215.52 ± 15.81 ^b^	295.21 ± 15.50 ^a^	198.32 ± 3.38 ^b^
Villus height: crypt depth, μm/μm	1.57 ± 0.10 ^a^	0.90 ± 0.04 ^b^	1.54 ± 0.14 ^a^
Villus width, μm	128.16 ± 5.05	127.87 ± 1.73	133.03 ± 6.67
Villus surface area, ×10^3^ μm^2^	67.73 ± 3.57 ^a^	54.40 ± 1.17 ^b^	66.25 ± 8.24 ^ab^

Results expressed as means and standard errors, *n* = 6. NC, piglets with normal birth weight fed a basal diet; IC, piglets with intrauterine growth retardation fed a basal diet; IR, piglets with intrauterine growth retardation fed a basal diet plus 300 mg/kg resveratrol. Significant differences between treatments are described by different letters in the form of superscripts, significant at *p* < 0.05.

**Table 4:** Effects of resveratrol on the mRNA expression of jejunal tight junction proteins in weaned piglets with intrauterine growth retardation.

Items	NC	IC	IR
*ZO1*	1.00 ± 0.28	0.64 ± 0.17	0.39 ± 0.15
*CLDN1*	1.00 ± 0.35	0.29 ± 0.08	0.29 ± 0.08
*CLDN2*	1.00 ± 0.08	0.77 ± 0.09	0.67 ± 0.12
*CLDN3*	1.00 ± 0.21	1.23 ± 0.23	1.73 ± 0.20
*OCLN*	1.00 ± 0.13 ^a^	0.41 ± 0.07 ^b^	0.35 ± 0.04 ^b^

Results expressed as means and standard errors, *n* = 5. NC, piglets with normal birth weight fed a basal diet; IC, piglets with intrauterine growth retardation fed a basal diet; IR, piglets with intrauterine growth retardation fed a basal diet plus 300 mg/kg resveratrol; *ZO1*, zonula occludens 1; *CLDN1*, claudin 1; *CLDN2*, claudin 2; *CLDN3*, claudin 3; *OCLN*, occludin. Significant differences between treatments are described by different letters in the form of superscripts, significant at *p* < 0.05.

**Table 5:** Effects of resveratrol on serum and jejunal redox status in weaned piglets with intrauterine growth retardation.

Items	NC	IC	IR
Serum			
MDA, nmol/mL	4.11 ± 0.53 ^b^	6.55 ± 0.56 ^a^	3.96 ± 0.31 ^b^
T-SOD, U/mL	105.53 ± 9.28 ^a^	49.02 ± 7.88 ^b^	107.76 ± 8.00 ^a^
GPX, U/mL	89.59 ± 4.14 ^a^	56.40 ± 5.25 ^b^	69.84 ± 9.67 ^ab^
GSH, μmol/L	1.67 ± 0.23	1.56 ± 0.41	2.29 ± 0.61
Jejunum			
MDA, nmol/mg protein	0.67 ± 0.10	1.00 ± 0.23	0.81 ± 0.10
T-SOD, U/mg protein	96.29 ± 4.16 ^b^	76.05 ± 4.93 ^c^	127.32 ± 3.86 ^a^
GPX, U/mg protein	43.54 ± 10.87	40.24 ± 3.19	42.44 ± 4.31
GSH, μmol/g protein	4.31 ± 0.33 ^ab^	3.14 ± 0.72 ^b^	6.27 ± 0.90 ^a^

Results expressed as means and standard errors, *n* = 6. NC, piglets with normal birth weight fed a basal diet; IC, piglets with intrauterine growth retardation fed a basal diet; IR, piglets with intrauterine growth retardation fed a basal diet plus 300 mg/kg resveratrol; MDA, malondialdehyde; T-SOD, total superoxide dismutase; GPX, glutathione peroxidase; GSH, reduced glutathione. Significant differences between treatments are described by different letters in the form of superscripts, significant at *p* < 0.05.

**Table 6:** Effects of resveratrol on jejunal mitochondrial energy metabolism in weaned piglets with intrauterine growth retardation.

Items	NC	IC	IR
ATP, nmol/g protein	254.17 ± 12.55 ^a^	113.13 ± 7.80 ^b^	219.49 ± 13.71 ^a^
Complex I, ng/mg protein	16.37 ± 1.33 ^a^	8.89 ± 0.31 ^b^	16.85 ± 1.37 ^a^
Complex III, ng/mg protein	11.18 ± 0.84	13.65 ± 2.49	9.86 ± 0.72
Complex V, ng/mg protein	4.77 ± 0.35	6.22 ± 1.41	4.82 ± 0.25

Results expressed as means and standard errors, *n* = 5. NC, piglets with normal birth weight fed a basal diet; IC, piglets with intrauterine growth retardation fed a basal diet; IR, piglets with intrauterine growth retardation fed a basal diet plus 300 mg/kg resveratrol. Significant differences between treatments are described by different letters in the form of superscripts, significant at *p* < 0.05.

## Data Availability

Data will be made available on request.

## Supplementary Materials

The following supporting information can be downloaded at: https://www.mdpi.com/article/10.3390/ani15030290/s1, Table S1: Composition and nutrient levels of the basal diet (air-dry basis).
