# Supplementary material for: Effects of Resveratrol on Redox Status, Jejunal Injury, and Mitochondrial Function in Intrauterine Growth-Retarded Weaned Piglets
Source: Animals (Basel). 2025 Jan 21;15(3):290. doi: 10.3390/ani15030290 (PMC11815716; doi:10.3390/ani15030290)
Supplement: Supplementary file 1 [file animals-15-00290-s001.zip › animals-3406150-supplementary.pdf]

Table S1. Composition and nutrient levels of the basal diet (air-dry basis)

Items	Contents, %
Corn	53.40
Flour	15.00
Soybean meal	17.50
Fermented soybean meal	2.50
Soybean oil	2.30
Fish meal	1.25
Soybean protein concentrate	2.50
Glucose	1.25
Phospholipid powder	1.00
limestone	0.91
Dicalcium phosphate	0.46
Sodium chloride	0.41
L-Lysine	0.42
DL-Methionine	0.10
Premix	1.00
Total	100.00
Calculated nutrient levels	
Digestible energy, MJ/kg	14.42
Crude protein, %	18.30
Ether extract, %	3.00
Crude fiber, %	6.00
Lysine, %	1.24
Calcium, %	0.78
Available phosphorus, %	0.38

Premix provided per kg of diet: vitamin A, 15000 IU; vitamin B1, 3 mg; vitamin B2, 6 mg; vitamin B6, 7 mg; vitamin B12, 0.03 mg; vitamin D3, 3200 IU; vitamin E, 22 mg; vitamin K3, 3 mg; niacin, 30 mg; pantothenic acid, 15.0 mg; folic acid, 1.20 mg; biotin, 0.08 mg; choline chloride, 500 mg; Fe, 120 mg; Cu, 120 mg; Zn, 110 mg; Mn, 43 mg; I, 0.70 mg; Se, 0.30 mg.
